# Inhibition of CCL3 abrogated precursor cell fusion and bone erosions in human osteoclast cultures and murine collagen-induced arthritis

**DOI:** 10.1093/rheumatology/key196

**Published:** 2018-07-20

**Authors:** Lauren A Jordan, Malin C Erlandsson, Benjamin F Fenner, Ruth Davies, Ann K Harvey, Ernest H Choy, Rachel Errington, Maria I Bokarewa, Anwen S Williams

**Affiliations:** 1 Division of Infection and Immunity, Cardiff, Wales, UK; 2 The Cardiff Regional Experimental Arthritis Treatment and Evaluation (CREATE) Centre, Cardiff, Wales, UK; 3 Department of Rheumatology and Inflammation Research, Institute of Medicine, Sahlgrenska Academy, The University of Gothenburg, Göteborg, Sweden; 4 Division of Cancer and Genetics, Cardiff University, School of Medicine, Cardiff, UK

**Keywords:** CCL3, macrophage inflammatory protein 1-alpha, osteoclast, collagen-induced arthritis, bone erosion

## Abstract

**Objective:**

Macrophage inflammatory protein 1-alpha (CCL3) is a chemokine that regulates macrophage trafficking to the inflamed joint. The agonistic effect of CCL3 on osteolytic lesions in patients with multiple myeloma is recognized; however, its role in skeletal damage during inflammatory arthritis has not been established. The aim of the study was to explore the role of osteoclast-associated CCL3 upon bone resorption, and to test its pharmacological blockade for protecting against bone pathology during inflammatory arthritis.

**Methods:**

CCL3 production was studied during osteoclast differentiation from osteoclast precursor cells: human CD14-positive mononuclear cells. Mice with CIA were treated with an anti-CCL3 antibody. The effect of CCL3 blockade through mAb was studied through osteoclast number, cytokine production and bone resorption on ivory disks, and *in vivo* through CIA progression (clinical score, paw diameter, synovial inflammation and bone damage).

**Results:**

Over time, CCL3 increased in parallel with the number of osteoclasts in culture. Anti-CCL3 treatment achieved a concentration-dependent inhibition of osteoclast fusion and reduced pit formation on ivory disks (*P* ⩽ 0.05). In CIA, anti-CCL3 treatment reduced joint damage and significantly decreased multinucleated tartrate-resistant acid phosphatase-positive osteoclasts and erosions in the wrists (*P* < 0.05) and elbows (*P* < 0.05), while also reducing joint erosions in the hind (*P* < 0.01) and fore paws (*P* < 0.01) as confirmed by X-ray.

**Conclusion:**

Inhibition of osteoclast-associated CCL3 reduced osteoclast formation and function whilst attenuating arthritis-associated bone loss and controlling development of erosion in murine joints, thus uncoupling bone damage from inflammation. Our findings may help future innovations for the diagnosis and treatment of inflammatory arthritis.

**Rheumatology key messages**

- Inhibition of CCL3 produced by human cells attenuated osteoclast differentiation and osteoclast-associated tissue resorption.
- The capacity of human osteoclasts to degrade bone tissue was unaffected by CCL3 antagonism.
- Pharmacological blockade of CCL3 during collagen-induced arthritis protected bones from erosion by reducing joint-associated osteoclasts.

## Introduction

A critical pathological feature of RA is the accumulation of immune cells in joints. Macrophages are one of the first cell types to infiltrate the inflamed synovia. They serve multiple effector functions (e.g. cytokine and chemokine production) and disrupt physiological bone remodelling [1]. Enrichment in macrophage-derived products is a characteristic feature of poor response to anti-rheumatic treatment [2, 3] and higher numbers of circulating monocytes predicted incomplete clinical response to MTX in untreated patients with RA [4]. Therefore, macrophages are good targets for the treatment of RA and such biomarkers can monitor clinical response.

Monocyte recruitment from the blood, their maturation into macrophages and differentiation into osteoclasts are vital in the advancement of RA [5]. These processes are regulated by chemokines [e.g. monocyte chemo-attractant protein 1 (CCL2), macrophage inflammatory protein 1-alpha (CCL3) and regulated upon activation, normal T cell expressed and secreted (CCL5)]. Such chemokines are induced during osseous inflammation [6–8] and are readily detected in sera and SF of RA patients with joint erosions [9, 10]. Collectively, these reports justify the potential of CC chemokine blockade for the inhibition of osseous inflammation and bone erosion during RA. Surprisingly few clinical trials have tested chemokine and chemokine receptor antagonism in treatment protocols for RA [11–13].

Localized osteolysis of articular bone and more generalized bone loss (osteopenia and osteoporosis) in the axial and appendicular skeleton are adverse characteristics of a more severe RA that is associated with a higher degree of functional disability and increased mortality [14, 15]. Bone resorption was thought to reflect the inflammatory burden in rheumatoid joints but recent evidence suggests the contrary. Biological anti-cytokine treatments (e.g. infliximab, etanercept, adalimumab, tocilizumab) revealed that inflammation could be uncoupled from progressive skeletal damage in RA and that bone erosion was not solely attributable to the action of TNF and IL-6 [16, 17]. Suppression of bone turnover through inhibition of RANK ligand (RANKL) prevented increase of erosions and had no evidence of an effect on activity measures of RA [18, 19]. Additionally, subclinical inflammation in synovia and tendons registered by MRI could cause erosion progress in absence of clinical signs of RA activity [19].

CCL2 [20], CCL3 [21] and CCL5 [22] are strongly linked with osteoclast differentiation from CD14-positive precursor cells arising from the monocyte–macrophage lineage. In addition, administration of a neutralizing anti-CCL2 antibody reduced ankle swelling in the rat CIA model [23], CCL3-deficient mice exhibited milder arthritis induced by an anti-type II collagen mAb [24] and treatment with polyclonal anti-CCL5 antibody ameliorated adjuvant-induced arthritis in rats [25]. Notably, the primary outcome for each study was focused upon the assessment of inflammatory indices. The impact of treatments on bone pathology was not determined. The assessment of chemokine function in these pre-clinical models of autoimmune arthritis supports the potential of chemokine-targeting therapies to modify disease progression and provided the rationale for this study.

Here, we challenged the notion that CCL3 controlled joint damage in arthritis solely by its pro-inflammatory action and hypothesized osteoclast differentiation to be the primary mode of action for CCL3. The aim of the study was to explore the role of CCL3 during differentiation of human osteoclasts upon resorption of ivory disks and to test the efficacy of pharmacological blockade of CCL3 on bone pathology in CIA. We identified CCL3 as a chemokine produced in the course of osteoclast maturation in direct proportion to bone erosion *in vitro*. An anti-CCL3 antibody potently inhibited resorption of ivory disks by osteoclasts without affecting CCL2, TNF or IL-6 levels in culture, and uncoupled bone resorption from inflammation. During CIA, inhibition of CCL3 protected bones against erosion by osteoclasts at a concentration that had no substantial effect on joint inflammation.

## Methods

### Reagents

Flow cytometry antibodies (Anti-human CD14-FITC and Isotype Control FITC) were purchased from eBiosciences (Altrincham, UK). For human osteoclast differentiation assays, M-CSF, RANKL, polyHistidine and anti-CCL3 antibody (MAB270; murine raised IgG1 reconstituted with sterile PBS) were sourced from R&D Systems, Abingdon, UK. Cell responses were compared using media supplemented with mouse IgG1 isotype as a control (MAB002, R&D Systems, Abingdon, UK). Commercially available ELISA kits [CCL2, CCL3, CCL5, IL-6, soluble IL-6 receptor (sIL-6R) and TNF-α] were run in accordance with the manufacturer’s instructions (R&D Systems, Abingdon, UK). Antibodies (R&D Systems, Abingdon, UK) for CIA [anti-CCL3 (MAB450) and isotype control (MAB006)] were reconstituted in sterile PBS.

### Animals

Male DBA/1 (Charles River, Margate, UK) mice were used for CIA assessments (ages 8–10 weeks at initiation). All procedures were approved by the Animal Welfare and Ethical Review Body at Cardiff University, under the authority of the Animal (Scientific Procedures) Act, 1986, and were performed in accordance with Home Office-approved license PPL 30/2928.

### Osteoclast differentiation assay

Human samples were collected under ethical approval to Cardiff Regional Experimental Arthritis Treatment and Evaluation (CREATE) (Arthritis Research UK grant Reference 2001^6^ to A.S.W. and E.H.C.). Peripheral blood mononuclear cells were isolated by Histopaque-1077 (Sigma Chemicals, Poole, UK) density gradient centrifugation from the venous blood of consenting healthy human volunteers (Research Ethics Committee for Wales Reference No. 12/WA/0045). Cells were washed three times with sterile HBSS (Life Technologies, Grand Island, NY, USA) and re-suspended in Roswell Park Memorial Institute medium 1640 (Life Technologies, Grand Island, NY, USA) supplemented with 10% heat-inactivated FBS (Biosera, Labtech International, UK), 20 mM l-glutamine, penicillin (50 U/ml) and streptomycin (50 μg/ml), henceforth called complete medium. Human monocytes and macrophages were positively selected from peripheral blood mononuclear cells using CD14 MicroBeads (MACS, Miltenyi Biotech, Bisley, UK). Flow cytometry confirmed ⩾95% cells were CD14 positive. Purified CD14-positive cells were re-suspended in complete medium (3.2 × 10^6^ cells/ml) and seeded onto ivory disks to model cell/substrate interactions on bone matrix [26]. CD14-positive cells were adhered to the ivory disks, which were subsequently transferred into individual wells containing 500 μl of complete medium containing M-CSF (10 ng/ml). After 7 days (day 0 of the osteoclast differentiation assay) the expanded population of CD14-positive osteoclast precursor cells (OCP) were cultured in complete medium containing M-CSF (5 ng/ml), RANKL (5 ng/ml) and polyHistidine (2.5 μg/ml). Antibodies (anti-CCL3 and IgG1; both at a final concentration of 8 ng/ml) were added on day 0 and replenished with each media change (days 3, 7, 10 and 14) when supernatants were collected. They were stored at −80°C until analysed by ELISA. Disks were harvested at each media change for time-course experiments or on day 14.

### Tartrate-resistant acid phosphatase stain for OCP and osteoclasts

Ivory disks were gently washed with PBS prior to cell fixation by glutaraldehyde solution for 15 min at 37°C. Osteoclasts were detected by tartrate-resistant acid phosphatase (TRAP) stain and haematoxylin was used as a counterstain to visualize nuclear material [27]. Cells were counted manually (Corel Paintshop Pro software, Corel Corporation, Ottawa, Canada) from images acquired by light microscopy (total disk coverage ⩾80%). Thirteen regions of interest were photographed per disk (Olympus Camedia C-3030 Digital Camera, Olympus UK & Ireland, Southend-on-Sea, UK). Total cells, TRAP-positive OCP and TRAP-positive osteoclasts [multinucleated cell (three or more nuclei) of ⩾30 μm diameter] were counted. A calibrated scale bar (ImageJ, National Institutes of Health, Bethesda, MD, USA) measured cell dimensions.

### Identification of resorption pits by toluidine blue staining

Disks were rendered cell-free by scraping with 70% alcohol, bleached by H_2_O_2_ (1% v/v) overnight, washed in water, stained with toluidine blue (0.5% w/v) for 60 s at room temperature and rinsed until water ran stain free [26, 28]. Pit borders were observed by light microscopy and recorded (four low-power regions of interest per disk; total coverage ⩾80%). The area of disk resorbed by osteoclasts was quantified using ImageJ software.

### Quantification of resorption pit parameters by confocal microscopy

Bleached disks were stained overnight with calcein (0.025% w/v) at room temperature, individually mounted (CyGel, Abcam, Cambridge, UK) and imaged by laser scanning confocal microscopy (Nikon Eclipse TE300, Nikon Instruments Europe BV, Amsterdam) linked to LaserSharp 2000 software. Optimized data collection parameters were kept constant for each disk (zoom: 1.5, pixels-by-lines: 512 × 512, step: 0.2 µm, xy pixel: 0.27 µm, objective: ×60 oil immersion 1.4 NA). 3D data (Z-stacks) were analysed by Metamorph (Molecular Devices, CA, USA) to quantify individual pit characteristics (lacuna area, perimeter, depth and volume). Pit parameters were measured in at least *n* = 2 disks per condition [volunteers (*n* = 8) matched for each group (anti-CCL3 *vs* IgG1)]. Comparable regions of interest (*n* = 10 per disk) were acquired by steering the field of view East to West and North to South via the centre point. Images were acquired every 10th frame, approximately. 3D topography of the disk surface was created with Fiji software (open source).

### Anti-CCL3 therapy in CIA

CIA was induced as previously described [29]. In brief, 2 mg/ml of chicken type II collagen (CII; Sigma-Aldrich, Gillingham, UK) was emulsified with an equal volume of complete Freund’s adjuvant. The emulsion (100 μl) was injected near the base of the tail intra-dermally on days 0 and 21. Mice were randomly assigned two treatment groups on day 21. Animals received five injections (100 μl) containing 5.0 mg/kg of either anti-CCL3 or isotype control administered intra-peritoneally on days 21, 23, 25, 27 and 28. Arthritis incidence and severity was assessed daily; the maximum arthritis severity was reached by one control mouse on day 29. Arthritis severity in each paw (paw score) was assessed using an established in-house scoring system: 0, normal; 1, mild but definite swelling in the ankle or wrist joint or redness and swelling limited to individual digits regardless of the number of digits affected; 2, moderate swelling of ankle or wrist; 3, severe redness and swelling of the ankle or wrist and proximal phalangeal joints; 4, maximally inflamed limb with involvement of multiple joints, no ankylosis; and 5, maximally inflamed limb with involvement of multiple joints, ankylosis present. The sum of scores for all four paws provided the clinical score for each mouse. Joint swelling was also measured by comparing calliper measurements of the hind paws (paw diameter).

### Histological assessment of CIA

Front limbs were fixed in formalin then decalcified in EDTA [27, 28]. Histological sections (7 μm) were cut from paraffin wax blocks, stained with haematoxylin and TRAP and scored using an established method. Synovial hyperplasia was scored 0–3, inflammatory cells within the synovial tissue were scored 0–5, inflammatory cells within the synovial cavity were scored 0–3 and articular cartilage/subchondral bone erosion was scored 0–3 [29, 30]. The composite score gave an arthritis index for each section. TRAP-positive osteoclasts were counted manually in joints of the elbow and wrist.

### X-ray for bone erosion quantification

Hind limbs were stripped of connective tissues and fixed in ethanol (70% v/v) prior to radiological assessments. Radiographs of the mouse paws were obtained using a Kodak In vivo Imaging System FX, and images were assessed using Kodak Molecular Imaging software (Kodak Molecular Imaging Systems, Connecticut, USA) as described previously [29]. A radiographic score was established for each limb by counting the number of eroded joints. The central/intermedium, fourth to fifth distal tarsals/fibulare, first to fifth metatarsal/tarsal and the first to fifth phalangeal/metatarsal joints were counted (score = 1 for each eroded joint and maximum score = 12 for each limb). Two independent musculoskeletal clinicians who were blinded to the CIA protocols scored the radiographs.

### Statistics

Sample size estimation for quantifying the difference between two groups was calculated from absolute values obtained from the first three osteoclast assays (G*Power version 3.0.10, University of Düsseldorf, Germany). Eight donors were required to achieve significance level of *P *= 0.05. Cells from each donor were tested in triplicate for each condition (mean value reported graphically). The total cell count for each disk in an individual well was used to calculate cytokine and chemokine concentration (picogram per millilitre) per well. Arthritis severity (by clinical score) from historic data was used to calculate sample size for CIA assessments; the minimum requirement to achieve significance level of *P *= 0.05 was *n* = 6 per group. Collated data for all donors and for CIA experiments are reported as mean (s.e.m.) unless stated otherwise. Analysis of variance was used to determine statistically significant differences between the means of three or more independent groups. Where Student’s *t*-test was used, the Bonferroni correction was applied to adjust the *P*-values when several dependent or independent statistical tests were performed simultaneously on a single data set. Spearman’s rank correlation coefficient was used to identify whether two variables were related in a monotonic function. Two-way analysis of variance was used to compare mean differences between groups by two independent variables (time and concentration) and by Chi-square, mid-P exact test. Analyses were performed using GraphPad Prism v4 (GraphPad Software, San Diego CA, USA), and the Open Source Epidemiologic Statistics for Public Health version 3.0 (http://www.openepi.com). *P* ⩽0.05 were considered significant.

## Results

### Supplementation of human OCP by RANKL increased CCL3 but not CCL2 or CCL5

**Fig. 1:**
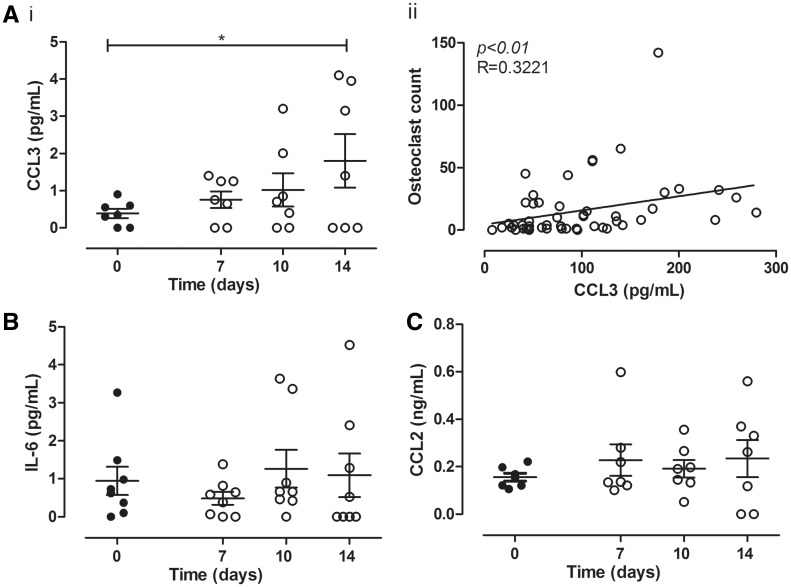
CCL3 was increased from OCP after RANKL supplementation Human CD14-positive monocytes cultured on bone substrate (*n* = 8 donors) were maintained in M-CSF (closed circles) or differentiated into osteoclasts (three or fewer nuclei) in M-CSF + RANKL medium (open circles). Media collected (days 0, 7, 10, 14) were analysed for CCL3 (**A**), IL-6 (**B**) and CCL2 (**C**) by ELISA. CCL3 increased significantly over the time-course [A (i)] and correlated significantly with osteoclast count [A (ii)]. IL-6 (B) and CCL2 (C) in M-CSF, or M-CSF + RANKL cultures, did not significantly change across the experimental time-course (one-way analysis of variance, post-Bonferroni). Mean value for each donor plotted. *P < 0.05, **P < 0.01. OCP = osteoclast precursor cells.

CC chemokines (CCL2, CCL3 and CCL5) and pro-inflammatory cytokines (IL-6 and TNF-α) were prospectively measured in the culture media collected while CD14-positive OCP differentiated into TRAP-positive OCP and multinuclear osteoclasts. A significant time-dependent increase in CCL3 levels was observed in cultures containing multinuclear osteoclasts but not in undifferentiated macrophages [Fig. 1A (i)]. There was a specific correlation between CCL3 concentration and formation of TRAP-positive multinucleated osteoclasts (*P *< 0.01). No such correlation was observed with total cell counts [Fig. 1A (ii)] or TRAP-positive OCP (data not shown). IL-6 (Fig. 1B) and CCL2 (Fig. 1C) were consistent across the time-course of the experiment and showed no correlation to the numbers of differentiated TRAP-positive OCPs. CCL5 and TNF-α levels were below the detection limit of for the ELISAs (30 pg/ml for CCL5 and 15 pg/ml for TNF-α).

### CCL3 correlates with bone resorption *in vitro*

**Fig. 2:**
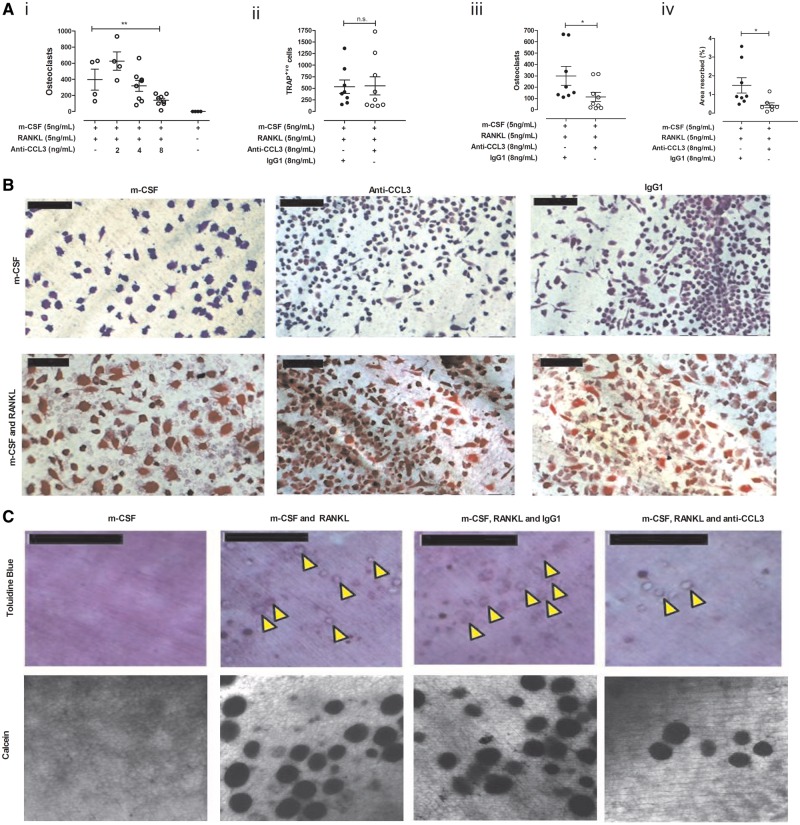
CCL3 correlates with bone resorption *in vitro* OCP (*n* = 8 donors, four replicates/condition) differentiated into osteoclasts in medium containing anti-CCL3 or IgG1 antibody. (**A**) Anti-CCL3 exerted a concentration-dependent inhibition on osteoclastogenesis (i), reducing total tartrate-resistant acid phosphatase (TRAP)-positive cells (ii), multinucleated osteoclasts (iii) and resorption (iv). (**B**) Disks stained with TRAP and haematoxylin (day 14). M-CSF cultures lacked osteoclasts (top), M-CSF + RANKL cultures showed strong TRAP staining (bottom; scale bar = 50 µm). (**C**) Resorption pits visualized by toluidine blue (top, scale bar = 250 μm) or calcein (bottom) reduced in anti-CCL3 (8 ng/ml) cultures *vs* IgG1 (yellow arrows). Mean value per donor plotted. *P ≤ 0.05, **P ≤ 0.01. OCP = osteoclast precursor cells.

In inflamed joints the autocrine effects of osteoclast-associated CCL3 are difficult to distinguish from the endocrine/exocrine effects of the chemokine. Concentration-dependent inhibition of osteoclastogenesis was observed using a CCL3 neutralizing antibody [Fig. 2A (i)] without affecting the overall number of adherent cells per disk. Formation of multinuclear TRAP-positive osteoclasts, but not TRAP-positive OCP, was inhibited by anti-CCL3 [Fig. 2A (ii) and (iii), and Fig. 2B]. Supplementation of M-CSF + RANKL cultures with IgG1 (8 ng/ml) had no significant effect on TRAP-positive OCP or TRAP-positive multinucleated osteoclast numbers. OCP cultured with M-CSF and anti-CCL3 or control IgG1 did not differentiate into TRAP-positive cells or multinucleated osteoclasts (Fig. 2B). Resorption of bone substrate was significantly reduced (*P *⩽ 0.05) by anti-CCL3 [Fig. 2A (iv)]. Results were consistent using two visualization methods namely, light microscopy and confocal microscopy (Fig. 2C).

### Resorption pit parameters were unaffected by CCL3 inhibition

**Fig. 3:**
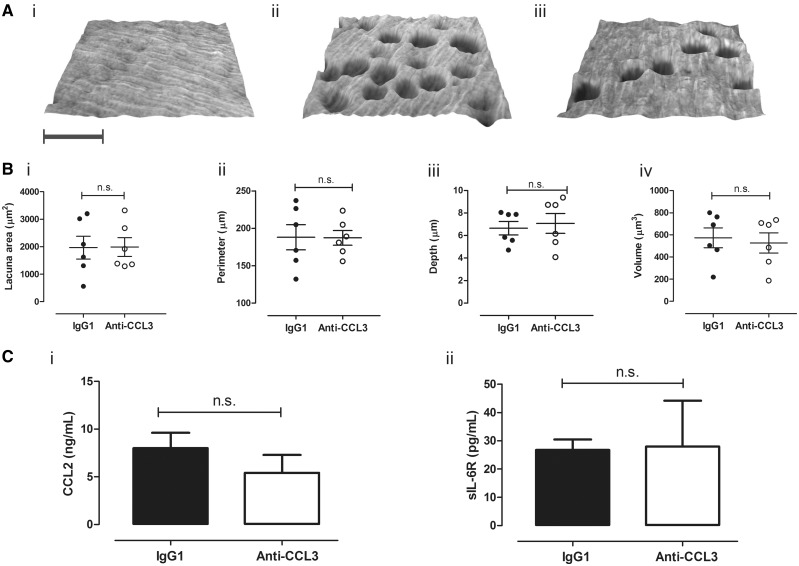
CCL3 inhibition had no effect on resorption pit parameters Calcein-stained ivory disks were imaged by fluorescence microscopy to quantify resorption pit parameters. (**A**) Representative topographical maps of M-CSF (i), M-CSF + RANKL + IgG1 (ii), M-CSF + RANKL + anti-CCL3 (iii) disks show their naturally undulating surface and resorption pits (spherical lacuna); scale bar = 40 μm. (**B**) Measured lacuna area (i), perimeter (ii), depth (iii) and volume (iv) for anti-CCL3 *vs* IgG1 were unchanged (8 ng/ml). (**C**) Levels of CCL2 (i) and sIL-6R (ii) were comparable in IgG1 and anti-CCL3 (8 ng/ml) cultures (day 14). Cells from healthy human volunteers (*n* = 6) were cultured, *n* = ≤2 disks/condition, mean (s.e.m.) for each donor plotted.

The diversity of multinucleated TRAP-positive osteoclast populations with respect to resorption and mobility is defined by their ability to create pits and trenches on a bone surface [31]. Topographical assessment of ivory disks by confocal microscopy [Fig. 3A (i–iii)] revealed pit characteristics indicative of stationary tissue resorption. Osteoclast migration tracks were seldom observed in cultures treated with anti-CCL3 or control antibodies. 3D measurements of individual resorption pit parameters demonstrated that anti-CCL3 treatment did not alter pit surface area, perimeter, depth or volume [Fig. 3B (i)–(iv)]. Finally, we measured levels of cytokines and chemokines with indirect effects on upon osteoclast-dependent disk erosion. On day 14, the levels of pro-osteoclastogenic IL-6, sIL-6R, CCL2 and CCL5 (only sIL-6R and CCL2 shown) were not significantly different in the cell cultures treated with anti-CCL3 compared with control cultures treated with M-CSF + RANKL only (Fig. 3C).

### Systemic inhibition of CCL3 reduced joint inflammation measured by histology

**Fig. 4:**
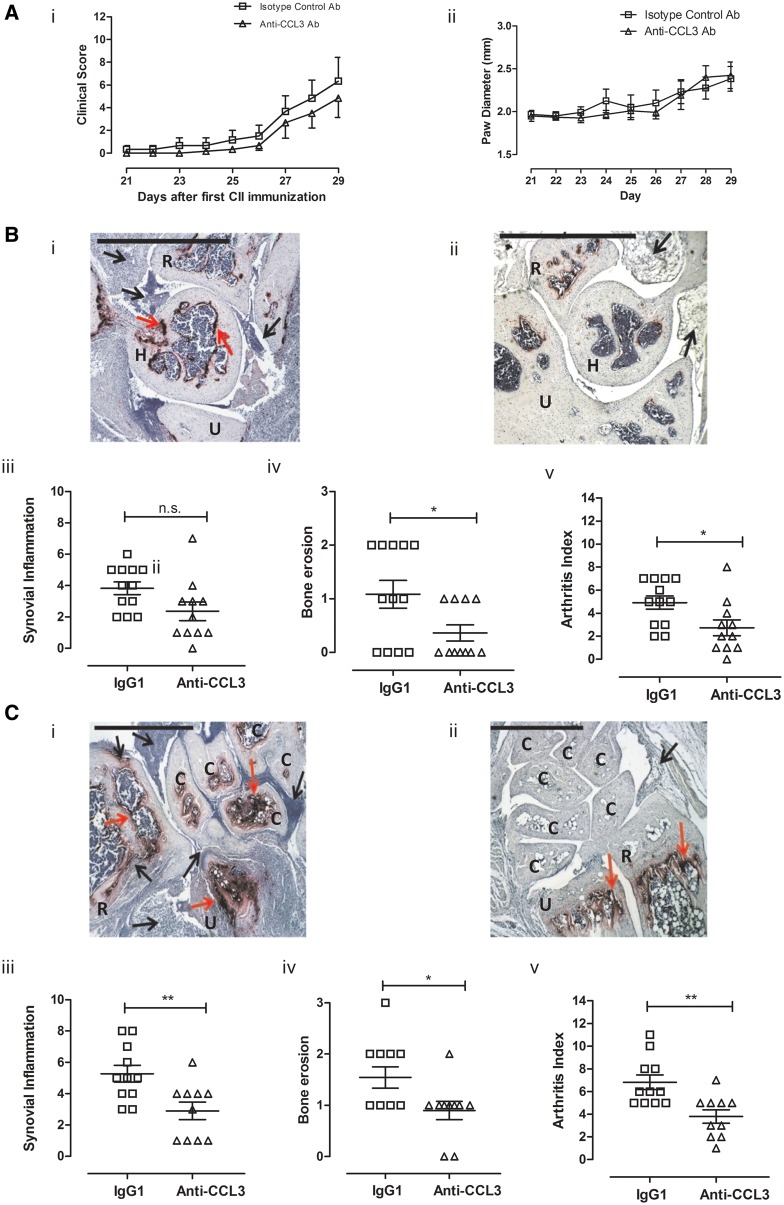
Systemic inhibition of CCL3 reduced histological joint swelling Mice with CIA received IgG1 or anti-CCL3 on days 21, 23, 25, 27 and 28 (5 mg/kg, *n* = 6/group). (**A**) Arthritis progression monitored by clinical scores (i) and paw diameter (ii) saw no statistical differences (two-way analysis of variance). (**B**) Representative tartrate-resistant acid phosphatase (TRAP) and haematoxylin-stained elbow joints from IgG1 (i) and anti-CCL3 (ii). Assessment of inflammation (iii), erosion (iv) and arthritic index (v). (**C**) IgG1 wrist histology showed intense TRAP-staining (i), which reduced in anti-CCL3 (ii), with significant reductions in inflammation (iii), erosion (iv) and arthritic index (v). H = humerus, U = ulna, R = radius, C = carpal. *P ≤ 0.05, **P ≤ 0.01, scale = 1 mm.

Diminutive, but not significant reductions in CIA-associated clinical score and paw diameter inflammation were observed in mice treated anti-CCL3 *vs* isotype control (Fig. 4A). Histological evaluation revealed a substantial reduction of leucocyte infiltration into synovial tissues, synovial hyperplasia and joint erosion by anti-CCL3 treated mice *vs* controls (Fig. 4B and C), resulting in a significantly reduced arthritis index in the wrist (*P *< 0.01) and elbow (*P *< 0.05) joint.

### CCL3 antagonism substantially reduced bone resorption in CIA

**Fig. 5:**
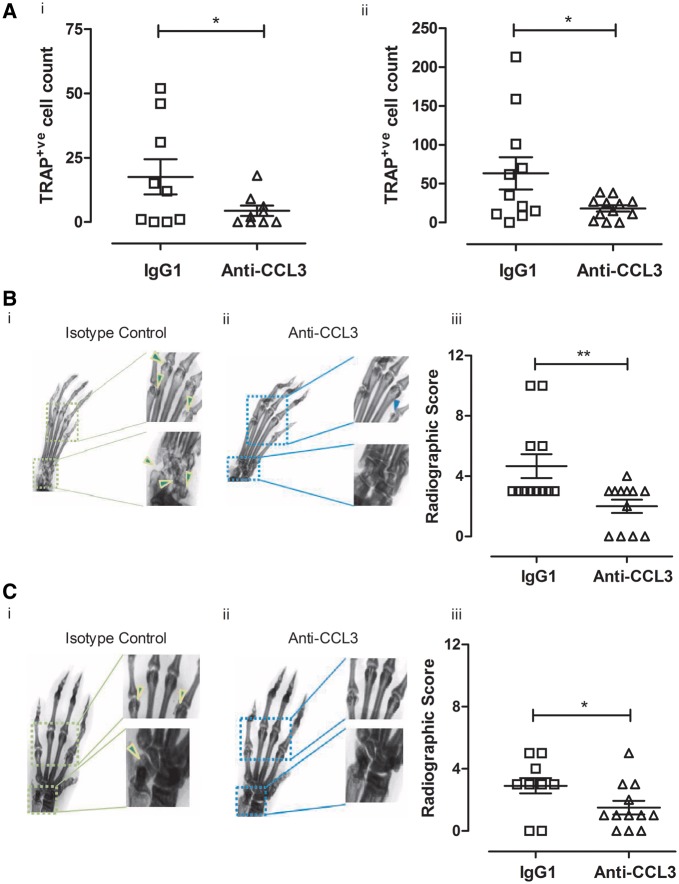
Bone erosions decreased after anti-CCL3 treatment during CIA Front and hind paws from CIA mice, treated with IgG1 or anti-CCL3, were processed for histology. (**A**) tartrate-resistant acid phosphatase (TRAP)-positive cells in the elbow (i) and wrist (ii) joints significantly reduced with anti-CCL3. (**B**) Radiographs of hind paws and (**C**) front paws were acquired from mice treated with IgG1 (i) or anti-CCL3 (ii) (*n* = 6 mice per group). Significant reductions in erosive radiographic score for in both hind [B (iii)] and front [C (iii)] paws were quantified in mice treated with anti-CCL3 compared with IgG1 controls. *P ≤ 0.05, **P ≤ 0.01.

CCL3’s role in modulating experimental arthritis has focused upon measuring synovitis and arthritis incidence rather than bone pathology [24]. Here, we present experimental evidence of the bone-protective effect of anti-CCL3 treatment during CIA in mice using two independent assessment methods. Firstly, histology showed that anti-CCL3 treatment significantly reduced TRAP-positive OCP in both the wrist (*P *< 0.05) and elbow (*P *< 0.05) joints compared with the control mice treated with IgG1 (Fig. 5A).

Secondly, analysis of radiographs identified significant differences in the extent of bone damage for both hind paws (*P *< 0.05) and fore paws (*P *< 0.01) in anti-CCL3-treated CIA mice *vs* controls (Fig. 5B and C). Notably, radiographic bone erosions were detected in the absence of visual signs of arthritis (arthritis score = 0), suggesting the uncoupling effects of anti-CCL3 treatment on bone resorption from inflammation. Only 8% of limbs from the control CIA mice were erosion free; the remainder had erosions in three or more joints. Twenty-one percent of limbs from anti-CCL3 treated mice were erosion free, and an additional 17% had fewer than three eroded joints. The number of erosion-free limbs was significantly higher in anti-CCL3- *vs* IgG1-treated mice (*P = *0.02).

## Discussion

CCL3 is a chemokine elevated in synovial fluid from patients with RA [32]. It is produced by several synovial cell-types including fibroblasts, macrophages and neutrophils [32–34]. Here, we emphasize a specific autocrine role of CCL3 in regulating osteoclast function, which has not been evaluated previously. We showed that inhibition of CCL3 using monoclonal antibodies strongly attenuated bone resorption by osteoclasts and arrested the development of bone erosion in CIA. This supports the notion that redressing the chemokine balance within the joint at an early stage of arthritis is critical for preventing irreversible bone damage and promoting a favourable outcome. In previously reported pre-clinical and clinical assessments, an inhibition of CCL3 binding to its receptor has been utilized [35–37] rather than inhibiting the chemokine itself.

We studied biosynthetic aspects of osteoclastogenesis and revealed CCL3 as the principle target for pharmacological intervention. CCL3 levels increased during osteoclast differentiation from human precursor cells and correlated with the number of multinuclear TRAP-positive osteoclasts but not with undifferentiated macrophages, suggesting that CCL3 has a specific impact upon the multinucleation phase of osteoclast differentiation. Divergent phase-specific effects for RANKL and M-CSF are also described: increased DNA synthesis and proliferation in the early stage and reduced proliferative activity in the latter half of osteoclast differentiation [38]. Here we show that pro-osteoclastogenic factors IL-6 and CCL2 were detectable in the culture media, but remain unaffected by either RANKL supplementation or CCL3 neutralization. In contrast to CCL3, their levels showed no correlation with osteoclast number. These results identify CCL3 as a priming agent for human osteoclast maturation.

The presence of osteoclast-originated CCL3 increased bone resorption whilst the inhibition of CCL3 prevented pit formation *in vitro*. Irrespective of whether osteoclast differentiation assays were conducted with or without anti-CCL3, the pits were remarkably consistent in their dimensions. This means that the resorptive function of the osteoclasts was unlikely to have been affected by CCL3 inhibition, showing opposing effects to TNF-α and RANKL [39]. It has been proposed that CCL3 initiates pre-osteoclast differentiation indirectly by enhancing RANKL expression in mixed cell cultures derived from rat bone marrow [40] and porcine marrow-derived cells [41]. Our findings present important evidence for a direct ability of osteoclast-produced CCL3 to coordinate and facilitate differentiation of new osteoclasts contributing to the bone microenvironment.

Cytokines like TNF-α and IL-6 are both pro-inflammatory and osteolytic [42, 43]; neutralization of CCL3 did change IL-6 production by human osteoclast *in vitro* and may explain the modest effect of CCL3 neutralization on signs of inflammation. Additionally, we were unable to detect a change in CCL3 in the plasma of CIA mice treated with anti-CCL3 antibodies (data not shown), supporting the notion that anti-CCL3 was working locally, and not systemically throughout the murine system.

Histological evaluation of joints revealed a significant reduction in erosive scores by the systemic administration of anti-CCL3 antibody and protected CIA joints from skeletal damage. This was contrary to the clinical CIA data where there was a small, but insignificant, reduction in clinical score and paw diameter by CCL3 blockade. Furthermore, X-ray analysis of joints revealed the presence of bone erosions in the absence of clinical signs of arthritis. CCL3 inhibition was initiated at a very specific time point when the formation of arthritogenic anti-collagen antibodies was complete and while clinical signs of arthritis were absent. To this end, the course of CIA showed similarity with individuals at an early pre-clinical phase of RA characterized by the production of antibodies against citrullinated peptides, which utilize osteoclasts to initiate a local influx of neutrophil to the joint via IL-8 [44, 45]. Our *in vivo* CIA data also support a role of CCL3 in leucocyte infiltration within the diseased joint, where leucocyte penetration into joint tissues was substantially reduced in anti-CCL3-treated mice. Hypothetically and in the correct environment, CCL3 could act in a similar way because it transforms into a neutrophil-attracting chemokine after cytokine or hormone priming [46–48].

### Conclusion

This study shows that CCL3 produced by osteoclasts is a potent regulator of bone resorption acting in the phase of osteoclast multi-nucleation. Targeted inhibition of CCL3 using monoclonal antibodies strongly suppressed bone formation of TRAP-positive osteoclasts and limited joint damage and bone erosions in the CIA model. Our observations enable us to propose a novel, autocrine control of bone environment and osteoclast differentiation by CCL3. An anti-CCL3 antibody has never been tested in a human RA trial. We present a justifiable case for the development of innovative pharmacological solutions to inhibit CCL3 for treating inflammatory arthritis.
